# Factors associated with health insurance enrolment among ghanaian children under the five years. Analysis of secondary data from a national survey

**DOI:** 10.1186/s12913-022-07670-7

**Published:** 2022-02-28

**Authors:** Emmanuel Anongeba Anaba, Akua Tandoh, Foday Robert Sesay, Theopista Fokukora

**Affiliations:** 1 Department of Population, Family and Reproductive Health, School of Public Health, University of Ghana, P. O. Box, L.G. 13, Legon, Ghana; 2 34 Military Hospital, Wilberforce, Freetown, Sierra Leone; 3 Department of Public Health, Faculty of Science and Technology, Cavendish University, P.O. Box 33145, Kampala, Uganda

**Keywords:** Health insurance, Children under five years, Ghana, Enrolment, Factors

## Abstract

**Background:**

Health insurance enrolment provides financial access to health care and reduces the risk of catastrophic healthcare expenditure. Therefore, the objective of this study was to assess the prevalence and correlates of health insurance enrolment among Ghanaian children under five years.

**Methods:**

We analysed secondary data from the 2017/18 Ghana Multiple Indicator Cluster Survey. The survey was a nationally representative weighted sample comprising 8,874 children under five years and employed Computer Assisted Personal Interviewing to collect data from the participants. In addition, Chi-square and Logistic Regression analyses were conducted to determine factors associated with health insurance enrolment.

**Results:**

The results showed that a majority (58.4%) of the participants were insured. Health insurance enrollment was associated with child age, maternal educational status, wealth index, place of residence and geographical region (*p* < 0.05). Children born to mothers with higher educational status (AOR = 2.14; 95% CI: 1.39–3.30) and mothers in the richest wealth quintile (AOR = 2.82; 95% CI: 2.00–3.98) had a higher likelihood of being insured compared with their counterparts. Also, children residing in rural areas (AOR = 0.75; 95% CI: 0.61–0.91) were less likely to be insured than children in urban areas.

**Conclusion:**

This study revealed that more than half of the participants were insured. Health insurance enrolment was influenced by the child's age, mother's educational status, wealth index, residence, ethnicity and geographical region. Therefore, interventions aimed at increasing health insurance coverage among children should focus on children from low socio-economic backgrounds. Stakeholders can leverage these findings to help improve health insurance coverage among Ghanaian children under five years.

## Background

Globally, protecting and improving the well-being of children under five years remains a public health priority. For instance, Target 3.2 of the Sustainable Development Goals seek to end preventable deaths of newborns and children under five years of age, with all countries aiming to reduce neonatal mortality and under five mortality by 2030 [1]. Evidence shows that under five mortality has declined over the last three decades worldwide. Between 1990 and 2019, the under five mortality rate has reduced by 59%, thus from 93 deaths per 1000 live births to 38 respectively [2]. Notwithstanding, the burden of under five mortality remains high. For example, in 2019 alone, about 14,000 children died every day before their fifth birthday worldwide. More than half (53%) of under five deaths in 2019 occurred in sub-Saharan Africa, with Nigeria and Somalia recording the highest under five mortality rates (117 deaths per 1000 live births) [2]. Most under five deaths are preventable with timely access to quality healthcare services and child health interventions [3]. Therefore, an integral recommendation—among many others—to accelerate progress towards reducing the mortality rate among children under five years is to ensure health equity through universal health coverage. So that all children can access essential health services without undue financial hardship [3]. Evidence shows that health insurance enrolment is linked to increased access and utilisation of healthcare services [4] and better health outcomes—particularly for maternal and child health [5–10].

In Ghana, the under five mortality rate was estimated to be 46 deaths per 1000 live births in 2019 [11], higher than the global rate of 38 deaths per 1000 live births. Financial constraints pose a significant barrier to accessing healthcare services, including child health services [12]. Therefore, under the National Health Insurance Act 650, the Government of Ghana established the National Health Insurance Scheme (NHIS) in 2003 to eliminate financial barriers to accessing health care [13]. Upon establishment, the scheme operated semi-autonomous Mutual Health Insurance Schemes in districts across the country. In 2012, a new law, Act 852, replaced Act 650. Under Act 852, all District Mutual Health Insurance Schemes were consolidated under a National Health Insurance Authority (NHIA) to ensure effective management and efficient service delivery [14]. The primary sources of financing the NHIS comprise a National Health Insurance Levy on selected goods and services, 2.5% contribution from the National Social Security Scheme by formal sector workers, individual premiums mainly from informal sector workers, and miscellaneous funds from investment returns, grants, donations and gifts from international donor partners and agencies [14].

Since its inception, Ghana’s NHIS has been considered one of Africa’s model health insurance systems. The benefit package of the NHIS covers the cost of treatment for more than 95% of the disease conditions in Ghana. The range of services covered includes but are not limited to outpatient care, diagnostic services, in-patient care, pre-approved medications, maternal care, ear, nose and throat services, dental services and all emergency services. Excluded in the NHIS benefit package are procedures such as dialysis for chronic renal failure, treatments for cancer (other than cervical and breast cancers), organ transplants and cosmetic surgery. Child immunization services, family planning and treatment of conditions such as HIV/AIDS and tuberculosis are also not covered. However, these services are provided under alternative government programs. Apart from pregnant women and children under five years, new members serve a waiting period of three months after registration before accessing health care under the scheme. Further, members of the scheme can access healthcare from health services providers accredited by the NHIA. These include public, quasi-government, faith-based, some but not all private health facilities and licensed pharmacies [15]. Despite the benefits of increased access to healthcare offered by NHIS membership and mandatory enrolment for all residents in Ghana, universal population coverage on the scheme has proved challenging. As of 2021, the NHIS had an active membership coverage of over 15 million people, equating to about 53% of Ghana’s estimated population [16]. However, a recent study examining NHIS enrolment within the last decade showed that, on average, only about 40% of all Ghanaians had ever registered with the Scheme [17]. That notwithstanding, utilization trends for in-patient and outpatient care at NHIS accredited health facilities continues to increase across the country [18, 19].

As part of efforts to increase coverage, a premium exemption policy for vulnerable populations, such as children under 18, was implemented. Thus, persons below 18 years are exempted from paying annual premiums [20] but must pay administrative charges, including the NHIS card processing fee [21]. Furthermore, in 2010, the National Health Insurance Authority decoupled children under five years from their parents’ membership. Therefore, children under five years can be active members of the NHIS even if their parents are not active members [22]. In addition, private health insurance schemes are emerging rapidly in Ghana, with premiums based on the calculated risk of subscribers.

The Ghana NHIS has been extensively investigated. Prior studies among the adult population showed that health insurance enrolment was associated with educational status, wealth, age, marital status, gender, type of occupation and place of residence [23–25]. In addition, an analysis of the 2011 Ghana Multiple Indicator Cluster Survey (MICS) revealed that a majority (73%) of children under five years were non-insured [26]. However, there is a paucity of literature on determinants of health insurance enrolment among children under five years. Therefore, this study aimed to determine factors associated with NHIS enrolment among children under five in Ghana using nationally representative survey data. Generating empirical evidence about factors influencing enrolment is essential to inform policy to help Ghana achieve Universal Health Coverage and Sustainable Development Goals.

## Methods

In this study, we analysed the 2017/2018 Ghana MICS [27]. The 2017/18 MICS collected demographic and health data across Ghana, including rural and urban settings. The sampling of participants was done in two phases. The first phase involved selecting 660 enumeration areas from 20 strata, proportional to size. The second involved the selection of 13,202 households within the selected enumeration areas. The weighted sample size of children under five years was 8,874. Ghana had ten administrative regions divided into 20 strata, of which ten are rural and ten are urban. Participants were selected across all the regions and strata. The inclusion criteria were under five children in the selected households or those who passed the night before the survey in the selected households. Data were collected using Computer Assisted Personal Interviewing (CAPI). The under five questionnaire was administered to caregivers of children below five years. Trained field officers and supervisors collected the data between October 2017 and September 2018. Details about the 2017/18 MICS are provided elsewhere [28].

The dependent variable in this study was health insurance status (i.e. is [*name*] covered by any health insurance?) coded as 1 = Yes and 0 = No. The independent variables identified in the literature included child and maternal characteristics. These include child’s age, maternal educational status, wealth index, ethnicity, geographic region and place of residence. Details about the coding are provided in Table 1. The complex nature of the survey was accounted for by employing the ‘svy’ STATA command. STATA/SE version 16 (StataCorp, College Station, Texas, USA) was used to analyse that data. Descriptive statistics were computed for participants’ characteristics and summarized in a table. The Chi-square test was employed to examine the association between participant characteristics and health insurance status at the bivariate level. Binary Logistic Regression was employed to identify significant predictors of health insurance enrolment among under five children. The results were reported at a 95% confidence level.

## Results

### Descriptive statistics

The results showed that a majority of the participants (58.4%) were insured, while a substantial minority (41.6%) were non-insured. Half of the participants were females (50.8%), and more than half (58%) were below 36 months. About three in ten (27.2%) mothers had no formal/pre-primary education, while 22.2% were in the poorest wealth quintile. Children from rural areas constituted 56.9%. In terms of ethnicity, 46.1% of the participants were Akan, 16.9% were Mole-Dagbani, and 37% were of other ethnic groups, such as Ewe and Gruma. Details are provided in Table 1.

**Table 1:** Socio-demographic characteristics of children, mothers and health insurance status in Ghana, 2017/18

**Characteristic**	***n***	**%**
**Sex of child**		
Male Female	4369 4505	49.2 50.8
**Age of child (months)**		
0–11 12–23 24–35 36–47 48–59	1700 1694 1750 1928 1802	19.2 19.1 19.7 21.7 20.3
**Mother's education**		
Pre-primary/none Primary Junior High School Senior High School Higher	2428 1790 3259 954 443	27.3 20.2 36.7 10.8 5
**Wealth quintile**		
Poorest Second Middle Fourth Richest	1966 1834 1769 1676 1630	22.2 20.7 19.9 18.8 18.4
**Residence**		
Urban Rural	3821 5053	43.1 56.9
**Region**		
Western Central Greater Accra Volta Eastern Ashanti Brong- Ahafo Northern Upper East Upper West	931 926 862 710 953 2111 833 1055 282 211	10.5 10.4 9.7 8 10.7 23.8 9.4 11.9 3.2 2.4
**Ethnicity**		
Akan Mole Dagbani Others (Ewe, Gruma etc.)	4091 1503 3280	46.1 16.9 37
**Health insurance status**		
Insured Non-insured	5186 3689	58.4 41.6

### Bivariate analysis

At the bivariate level, child age was significantly associated with health insurance enrolment (*p* < 0.05). Also, mothers’ education, wealth quintile, residence, geographic region, and ethnicity were significantly associated with health insurance enrollment (*p* < 0.05) among children under five years. A majority (56.1%) of children aged 0–11 months were not insured with the National Health Insurance Scheme. Less than half of children in the Central Region were insured, while eight in ten children were insured in the Brong-Ahafo Region. Details are provided in Table 2.

**Table 2:** Association between participant characteristics and health insurance status

**Characteristic**	***n***	**Non-insured** **(%)**	**Insured** **(%)**	**Chi-square**	***p***-value
**Sex of child**					
Male Female	4369 4505	41.3 41.8	58.7 58.2	0.2961	0.7311
**Age of child (months)**					
0–11 12–23 24–35 36–47 48–59	1700 1694 1750 1928 1802	56.1 43.6 34.6 37.7 37	43.9 56.4 65.4 62.3 63	29.3826	< 0.0001
**Mother's education**					
Pre-primary/none Primary Junior High School Senior High School Higher	2428 1790 3259 954 443	42.6 49.4 41.5 34.5 20.1	57.4 50.6 58.5 65.4 79.9	149.8059	< 0.0001
**Wealth quintile**					
Poorest Second Middle Fourth Richest	1966 1834 1769 1676 1630	48.7 44.4 42.6 41.1 29.1	51.3 55.6 57.4 58.9 70.9	152.59	< 0.0001
**Residence**					
Urban Rural	3821 5053	35.8 45.9	64.2 54.1	92.0315	< 0.0001
**Region**					
Western Central Greater Accra Volta Eastern Ashanti Brong- Ahafo Northern Upper East Upper West	931 926 863 710 953 2111 833 1055 282 211	46.7 53 49.7 42.5 39.3 41.9 22.6 42.1 24.1 34.1	53.3 47 50.3 57.5 60.7 58.1 77.4 57.9 75.9 65.9	250.9397	< 0.0001
**Ethnicity**					
Akan Mole Dagbani Other (Ewe, Gruma etc.)	4091 1503 3280	42.2 38.5 42.3	57.8 61.5 57.7	7.3800	0.3330

### Multivariable analysis

At the crude analysis level, health insurance enrolment was significantly predicted by wealth quintile, child’s age, mother’s education, place of residence, geographical region and ethnicity (*p* < 0.05). At the adjusted analysis level, it was found that children in the poorest wealth quintile were less likely to be insured compared with children in the second (AOR = 1.47; 95% CI: 1.15–1.89), middle (AOR = 1.59; 95% CI:1.16–2.17), fourth (AOR = 1.73; 95% CI:1.28–2.35) and richest (AOR = 2.82; 95% CI: 2.00-3.98) wealth quintiles. Children aged 0–11 months were less likely to be insured compared with children aged 12–23 months (AOR = 1.72; 95% CI: 1.42–2.10). Children whose mothers had higher education were two times (AOR = 2.14; 95% CI: 1.39–3.30) more likely to be insured compared with children whose mothers had no formal education. In addition, children in rural areas (AOR = 0.75; 95% CI: 0.61–0.91) had lesser odds of being insured compared with children in urban areas. Also, children in the Northern Region (AOR = 3.23; 95% CI: 2.18–4.80), Upper West Region (AOR = 4.82; 95% CI: 3.04–7.66) and Upper East Region (AOR = 8.74; 95% CI: 5.35–13.40) had a higher probability of being insured compared with children in the Greater Accra Region. Details are provided in Table 3.

**Table 3:** Logistic Regression on predictors of health insurance status among children under five years in Ghana, 2017/18

**Covariate/exposure**	**Crude analysis** **OR (95% CI)**	**Wald** ***p***-value	**Adjusted analysis** **OR (95% CI)**	**Adjusted Wald** ***p*** **-value**
**Sex of child**				
Male Female	1(ref) 0.98 (0.85–1.12)	0.7311	1 (ref) 0.98 (0.85–1.13)	0.8045
**Age of child (months)**				
0–11 12–23 24–35 36–47 48–59	1(ref) 1.66 (1.37–2.00) 2.42 (2.02–2.89) 2.12 (1.76–2.55) 2.17 (1.79–2.63)	< 0.0001	1 (ref) 1.72 (1.42–2.10) 2.70 (2.22–3.29) 2.30 (1.88–2.81) 2.44 (1.99–2.99)	< 0.0001
**Mother’s education**				
Pre-primary/none Primary Junior High School Senior High School Higher	1(ref) 0.76 (0.62–0.93) 1.05 (0.87–1.26) 1.40 (1.09–1.81) 2.96 (2.07–4.25)	< 0.0001	1(ref) 0.83 (0.67–1.02) 1.16 (0.95–1.42) 1.37 (1.02–1.82) 2.14 (1.39–3.30)	0.0002
**Wealth quintile**				
Poorest Second Middle Fourth Richest	1(ref) 1.19 (0.95–1.49) 1.28 (0.98–1.66) 1.36 (1.05–1.76) 2.31 (1.77–3.02)	< 0.0001	1(ref) 1.47 (1.15–1.89) 1.59 (1.16–2.17) 1.73 (1.28–2.35) 2.82 (2.00–3.98)	< 0.0001
**Residence**				
Urban Rural	1(ref) 0.66 (0.55–0.79)	< 0.0001	1(ref) 0.75 (0.61- 0.91)	0.0002
**Region**				
Greater Accra Western Central Volta Eastern Ashanti Brong- Ahafo Northern Upper East Upper West	1(ref) 1.13 (0.77–1.63) 0.87 (0.60–1.26) 1.33 (0.83–2.13) 1.52 (1.06–2.18) 1.37 (0.99–1.89) 3.38 (2.27–5.04) 1.36 (0.97–1.90) 3.14 (2.21–4.44) 1.92 (1.33–2.76)	< 0.0001	1(ref) 1.93 (1.32–2.80) 1.38 (0.92–2.08) 2.81 (1.65–4.80) 2.68 (1.86–3.86) 2.13 (1.50–3.02) 6.61 (4.24–10.30) 3.23 (2.18–4.80) 8.74 (5.35–13.40) 4.82 (3.04–7.66)	< 0.0001
**Ethnicity**				
Akan Mole Dagbani Others (Ewe, Gruma etc.)	1(ref) 1.17 (0.94–1.45) 1.00 (0.83–1.19)	< 0.0001	1(ref) 1.07 (0.80–1.43) 1.09 (0.90–1.31)	< 0.0001

## Discussion

The findings showed that more than half (58.4%) of the participants were covered by health insurance. Thus, caregivers/parents of insured children were protected against out-of-pocket payment, which is a risk factor for catastrophic health care expenditure and poverty [29]. A similar study revealed that 57% of Ghanaian children below 18 years were covered by health insurance [21]. Another household survey across three districts in Ghana reported that 55.9% of the participants were insured. A similar nationally representative survey demonstrated that 66% and 52.6% of women and men aged 15–49 years were insured respectively [30].

Further, our finding is similar to a study in Shanghai, China, where 56.5% of children under eight years were covered by health insurance [31]. However, health insurance coverage in this study was higher than coverage in other African countries. For instance, an analysis of data from four African countries revealed that Ghana had the highest health insurance coverage of 62.4% and 49.1% for females and males respectively. Followed by Kenya (18.2% for females and 21.9% for males), Tanzania (9.1% for females and 9.5% for males) and Nigeria (1.1% for females and 3.1% for males) [32]. The difference in findings may be attributed to contextual factors and health insurance policies. For instance, Ghana’s National Health Insurance Scheme (NHIS) covers more than 95% of the disease conditions in the country, including medications, medical investigations, outpatient and in-patient services. Also, women who register with NHIS have access to free maternal health services, such as antenatal, delivery and postnatal services. Children under 18 years, indigents, the elderly, persons with disability or mental disorders, Social Security and National Insurance Trust (SSNIT) contributors and pensioners are exempted from paying premiums but must renew their membership every year [33].

In addition, we found that four in ten children were not covered by health insurance. Therefore, parents/caregivers of uninsured children would have to pay-out-of pocket when accessing child health care services. Out-of-pocket payment has the potential of putting caregivers at risk of catastrophic healthcare expenditure and poverty. Also, parents of non-insured children are more likely to postpone or delay seeking health care, hence putting the child’s life at risk of poor health outcomes [34]. This finding is similar to findings from previous studies in Ghana and elsewhere. For instance, a study revealed that 43.2% of Ghanaian children under eighteen years were uninsured [21]. Another study among children under seven years in Shanghai, China, reported that 43.5% of the participants were uninsured [31]. This finding may be explained by individual, financial, country-specific and health system-related factors [22]. In Ghana, children under five years are exempted from paying NHIS premiums. However, they must pay membership card processing fees and renewal fees every year [33]; hence, it may pose a barrier for their caregivers.

Furthermore, recent evidence shows that persons insured with Ghana’s NHIS still pay out-of-pocket for services in accredited health facilities [35]. Reasons for non-registration or non-renewing of membership with the NHIS include financial constraints, lack of confidence in the scheme, dissatisfaction with services, shortage of insured medications, long waiting time, payment of illegal charges and non-use of health services [36]. Going forward, the Ministry of Health, National Health Insurance Authority, Ministry of Gender, Children and Social Protection and health providers would have to collaborate to improve health insurance coverage for Ghanaian children under five years. However, there is a need for empirical evidence on the correlates and reasons for non-enrolment among children under five years. Hence, we recommend that future studies should explore these grey areas.

In addition, the findings revealed that health insurance enrolment was influenced by child’s age, mother’s educational status, wealth index, region, ethnicity and place of residence. Children whose mothers were less educated had low likelihoods of being insured. A similar study found that well-educated mothers were more likely to enroll their children on health insurance [37]. Another study in Shanghai, China, showed that children of women with low education were less likely to be covered by health insurance [31, 32]. A probable explanation is that less educated mothers may lack adequate understanding of the health insurance process and the benefits package due to their inability to access information [5]. Evidence shows that Ghanaian women who had access to information were more likely to be insured [38]. Women with higher educational status are more empowered to make health-seeking decisions. Also, children from wealthy families were more likely to be covered by health insurance. Previous studies have supported this finding [39]. Another study in Shanghai, China, revealed that children from the lowest income households had lesser odds of being insured [31]. A possible explanation is that wealthy parents/caregivers have large disposable incomes. Hence, they can afford health insurance premiums, NHIS card processing charges, and annual renewal fees. It implies that the purpose of the NHIS as a pro-poor social intervention has not been well achieved.

Also, children in rural areas had lower chances of being insured. A study in Ghana reported that women living in remote settings had lower odds of insurance coverage than those staying in urban areas [37]. A conceivable explanation for this finding is that parents of children residing in urban areas may have easy access to health insurance offices. In Ghana, few NHIS offices are sited in rural areas, leading to delays in registering and printing insurance cards. There are also few NHIS personnel and logistics in the rural areas compared to the urban areas [36]. These factors may explain the disparities in insurance coverage across the place of residence. It was revealed that children from the nine other administrative regions were more likely to be insured than those from the Greater Accra Region, Ghana’s capital city. A similar study reported that children in the Greater Accra region were more likely to be non-insured compared with the other regions in Ghana [21]. The Greater Accra region has the lowest health insurance coverage in Ghana [40].

Moreover, we found that children from regions with a high incidence of poverty were more likely to be insured. This finding was expected because the poor perceive health insurance as a form of social security that protects them against catastrophic health care expenditure during health emergencies [41]. This finding may explain why the poorest region in Ghana (Upper East region) has the highest NHIS coverage [40]. Additionally, health insurance enrolment was associated with child’s age. Thus, children aged twelve months or older were more likely to be insured. A similar study in Shanghai, China, reported that older children were less likely to be uninsured [31]. The Free Maternal Health Policy may explain this finding. In Ghana, pregnant women who register with the NHIS have free maternal health care services up to three months postpartum [42]. Our findings imply that vulnerable children did not have health insurance. Consequently, their caregivers/parents may be predisposed to catastrophic health care expenditure. Besides, evidence shows that uninsured children are predisposed to poor health outcomes [43]. Therefore, in the quest to increase health insurance coverage, future interventions should prioritize children from the low socio-economic background.

### Strengths and limitations of the study

One major strength of this study is that we analysed nationally representative data so the findings from this study can be generalized to the population. This study is one of the few studies in Ghana investigating socio-demographic determinants of child health insurance. However, this study is not devoid of limitations. Cross-sectional studies cannot establish causal relationships, so the findings should be interpreted with caution. In addition, health insurance status was self-reported by caregivers/parents of the children. Therefore, it may be subjected to social desirability or recall biases.

## Conclusions

This study demonstrated that more than half of the children were covered by health insurance. Health insurance enrolment was associated with wealth index, mother’ educational status, child’s age, type of residence, geographical region and ethnicity. Policymakers can leverage these findings to help improve health insurance coverage for children in Ghana. Interventions that seek to improve health insurance coverage must prioritize children from poor socio-economic backgrounds. Future studies should employ qualitative designs to expose the many intricate views of caregivers regarding health insurance enrolment among children under five years. Also, further studies may explore factors associated with non-enrollment among children under five years.

## Data Availability

The data used in this study is owned by UNICEF, therefore, the authors cannot share the data. Interested persons can contact UNICEF for the data (contact via accra@unicef.org). The authors confirm they did not have any special access or privileges to the data that other researchers would not have.

## Abbreviations

NHIS: National Health Insurance Scheme
MICS: Multiple Indicator Cluster Survey
SDG: Sustainable Development Goals
UNICEF: United Nations International Children’s Emergency Fund
CAPI: Computer Assisted Personal Interviewing
